# V(D)J Recombination and the Evolution of the Adaptive Immune System

**DOI:** 10.1371/journal.pbio.0000016

**Published:** 2003-10-13

**Authors:** Eleonora Market, F. Nina Papavasiliou

## Abstract

In order for the immune system to generate its vast numbers of receptors, B- and T-cell receptor genes are created by recombining preexisting gene segments. This well- coordinated set of reactions is explained

The immune system needs to be able to identify and ultimately destroy foreign invaders. To do so, it utilizes two major types of immune cells, T cells and B cells (or, collectively, lymphocytes). Lymphocytes display a large variety of cell surface receptors that can recognize and respond to an unlimited number of pathogens, a feature that is the hallmark of the “adaptive” immune system. To react to such a variety of invaders, the immune system needs to generate vast numbers of receptors. If the number of different types of receptors present on lymphocytes were encoded by individual genes, the entire human genome would have to be devoted to lymphocyte receptors. To establish the necessary level of diversity, B- and T-cell receptor (BCR and TCR, respectively) genes are created by recombining preexisting gene segments. Thus, different combinations of a finite set of gene segments give rise to receptors that can recognize unlimited numbers of foreign invaders. This is accomplished by a supremely well-coordinated set of reactions, starting with cleaving DNA within specific, well-conserved recombination signal sequences (RSSs). This highly regulated step is carried out by the lymphocyte-specific recombinationactivating genes (*RAG1* and *RAG2*). The segments are then reassembled using a common cellular repair mechanism.

For foreign invaders and their proteins (antigens) that are not part of the host to elicit an immune response, the immune system must be able to recognize countless numbers of antigens. For obvious reasons, an unlimited number of unique antigen receptors cannot be genetically encoded. Rather, the necessary diversity in receptors is achieved by creating variations in the antigen-recognition regions of the receptors of both B cells and T cells. These regions are created by the pairing of two different protein segments, called polypeptide chains (heavy [H] and light [L] chains in the case of the BCR and α and β chains in the case of the TCR), which form a cleft that provides a binding site for the antigen. The mechanism that generates variation in the antigen-binding pockets of these receptors involves mixing and matching variable (V), diversity (D), and joining (J) gene segments in a process called V(D)J recombination. To assemble a single functional receptor, preexisting V, D, and J gene segments are rearranged to yield a contiguous V(D)J region, just upstream of another element of the receptor, the constant (C) region (Figure 1A).

**Figure 1:**
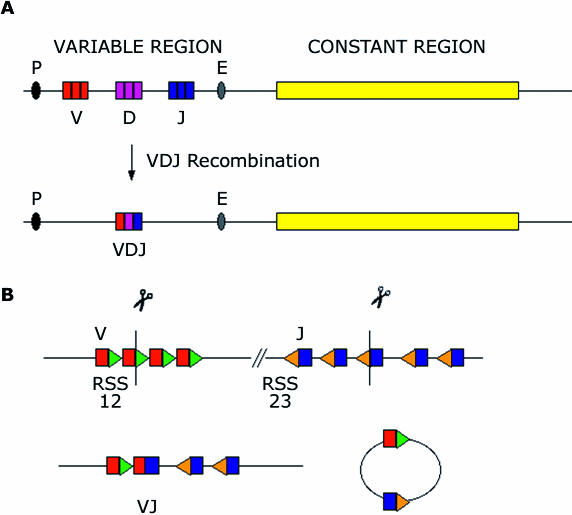
V(D)J Recombination Takes Place within the BCR and TCR Loci (A) Schematic of a receptor locus.V, D, and J segments are found just upstream of the constant region. (B) A cartoon view of a VJ recombination reaction. V segments (red) are flanked by RSSs with 12 bp-long spacers (green), while the J segments are flanked by RSS with 23 bp-long spacers (orange). Breaks are introduced directly between the heptamer and the coding sequence, and a CJ is formed between a V and a J segment, while the RSS ends are put together to form an SJ within a circular DNA that is later lost. Symbols: P, promoter; E, enhancer.

The BCR H chain and the TCR β chain consist of V, D, and J segments, while BCR L chains and the TCR α chain are comprised of only V and J segments. The number of each type of segments within the chains allows for a large but finite combinatorial possibility in rearrangement, a phenomenon termed combinatorial diversity (Table 1). However, variations generated by V(D)J recombination are uncountable because they do not simply rely on the number of gene segments. Further diversity is introduced because the junctions between rearranged gene segments contain small insertions and deletions (junctional diversity). Finally, both BCR and TCR are heterodimers (consisting of two unmatched polypeptides), so the possibilities of different pairing between the chains can also increase variation. Successful V(D)J rearrangement is clearly useful in terms of antigen recognition, and it is absolutely required for the development and survival of B and T cells.

**Table 1:**
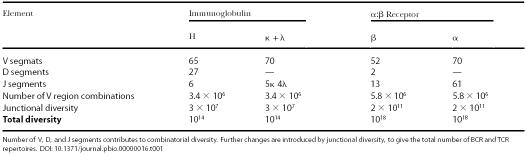
Diversification of BCRs and TCRs

Number of V, D, and J segments contributes to combinatorial diversity. Further changes are introduced by junctional diversity, to give the total number of BCR and TCR repertoires

## V(D)J Recombination: A Cut-and-Paste Reaction

In the first part of the “cut-and-paste” reaction, breaks within both strands of the DNA helix (double-stranded breaks) are made within the RSS sites; in the second part, the newly created breaks are repaired by the cell's general DNA repair pathway. In the initial phase, two lymphocyte-specific proteins that are encoded by the recombinationactivating genes (*RAG1* and *RAG2*) work together to recognize and bind RSSs. The complex consisting of these two proteins, RAG1–RAG2 (henceforth RAG), cuts the DNA between the rearranging DNA segments and the adjacent RSS motifs (Figure 1B). The second step of the reaction glues together the ends of the chromosome containing the rearranging segments, which will ultimately code for the receptor and are called coding joints (CJs). The portion of DNA between the rearranged segments is shed from the genome, but it too gets glued together in a minicircle (a signal joint [SJ]). Typically, SJs are rapidly and precisely fused, but CJs are ligated more slowly, in part because their fusion is not precise—small insertions are present quite often, and even deletions can be detected. (For detailed reviews, see Fugmann et al. 2000; Gellert 2002). Both pasting reactions are necessary for creating the receptors as well as for preventing havoc within the genome.

## RAGs: Indispensable for V(D)J Recombination

RAG proteins carry out the first enzymatic step of the reaction—site-specific cleavage of DNA (van Gent et al. 1995). Artificial expression of RAGs in mammalian cells other than B- or T-lymphocytes suggests that RAG is the only lymphocyte-specific factor required for this recombination event to occur (Schatz and Baltimore 1988). Indeed, in mice whose *RAG* genes have been deleted (*RAG* ^−/−^), V(D)J recombination is completely abolished, and these mice have neither mature B nor T cells (Mombaerts et al. 1992; Shinkai et al. 1992). A similar type of immunodeficiency, called Omenn syndrome, is seen in people with mutations in their *RAG* genes (Villa et al. 1998).

In test-tube experiments, purified RAG proteins are sufficient for cleavage of a synthetic DNA containing the appropriate RSS (McBlane et al. 1995). This reaction can be subdivided into two stages. First, a nick is made in the DNA, at a specific site within the RSS, leaving specific chemical modifications at the ends (Figure 1B). Then, one free end with a specific (3′-hydroxyl) group forms a new chemical (diester) bond with a different chemical (phosphoryl) group on the complementary nucleotide of the opposite strand (this is called a transesterification reaction). This results in the formation of a hairpin at the coding end, while leaving the signal end blunt (McBlane et al. 1995).

## RSSs: The Targets of the Reaction

**Figure 2:**
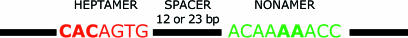
RSSs Consist of a Fairly Conserved Heptamer and Nonamer Sequence, Separated by a Spacer Element Heptamer is shown in red and nonamer in green. Conserved nucleotides are shown in bold. The spacer is either 12 or 23 bp long.

RSSs are found next to every variable (V), diversity (D), and joining (J) segment. They consist of three distinct elements: a heptamer and a nonamer sequence, separated by a spacer element—either 12 or 23 bp long (Figure 2) (Tonegawa 1983; Akira et al. 1987). Although the two RAG proteins work together in a protein complex, they do have unique functions. RAG1 binds both the 12- and 23-RSSs with equal affinities, while RAG2 does not bind either RSS sequence. This suggests that RAG1 forms the initial complex with DNA, which then recruits and is stabilized by RAG2 (Fugmann et al. 2000).

Both the heptamer and the nonamer contain nucleotides that are absolutely required for efficient V(D)J recombination. The first three nucleotides within the heptamer are conserved, whereas mutations in the next four positions affect but do not abolish the reaction. Within the nonamer, positions 5 and 6 are conserved, while variations are tolerated elsewhere in the sequence (Figure 2). The length of the spacer (but not its sequence) was thought to play an important role in regulating which elements could be recombined (Gellert 2002): a 12mer differs from a 23mer by a single turn of the DNA helix, providing for the proteins that bind RSSs to remain in the same rotational phase (Gellert 2002). The paper in this issue of *PLoS Biology* by Lee et al. (2003) now challenges this paradigm. They report that not only the length of the spacer but also its sequence are important, so that changes in the spacer sequence directly affect rates of recombination. Based on these data, they have developed an algorithm that accurately predicts the relative efficiencies of RSS binding, cleavage, and rearrangement based on nucleotide sequence.

## Breaks: Necessary Precursors of Recombination

After RAG cuts the DNA at the RSS, the two sides of the break are different. The coding ends are closed to resemble hairpins, while the RSS ends are open and blunt. These blunt RSS ends are rejoined rapidly, forming SJs, but before the coding ends can be fused, the hairpins must be opened (Gellert 2002).

Normally, hairpins are opened either at the tip or on the side. In either case, an enzyme called terminal deoxynucleotidyl transferase can add a small amount of random (nontemplated) nucleotides to freed ends, and this phenomenon of N-nucleotide addition contributes to junctional diversity of the CJs (Bassing et al. 2002).

When the hairpin is opened on the side, the reaction leaves a short overhang on one strand. When the overhangs are filled, palindromic sequences are generated, and these modifications are referred to as “P-nucleotides” (Bassing et al. 2002). These, too, contribute to the diversity of the CJs and ultimately, when the rearranged gene produces protein, to more variability in the antigen-binding pocket of the receptor.

## Resolution: The Final Step

As mentioned, *RAG* ^−/−^ mice do not have mature B and T cells. This causes a severe combined immunodeficiency syndrome (SCID) characterized by a complete block in B- and T-cell development, but no other defects (Mombaerts et al. 1992; Shinkai et al. 1992). However, there are other molecular deficiencies that also have a SCID phenotype. One of these is mapped to the enzyme DNA protein kinase (DNA-PKcs), which is required for the proper joining of DNA ends (Bosma et al. 1988). Mice deficient in DNA-PKcs can initiate V(D)J recombination, but cannot form the CJs (Gao et al. 1998). These mice are also sensitive to processes that induce DNA double-stranded breaks, such as ionizing radiation (Gao et al. 1998). Hence, the repair pathway responsible for fixing DNA breaks caused by radiation also creates CJs. Indeed, along with DNA-PKcs, other proteins of this nonhomologous end-joining repair pathway are important for the completion of V(D)J recombination (such as Ku70 and Ku80, Artemis, XRCC4, and DNA ligase IV) (Bassing et al. 2002).

## An Evolutionary Model of V(D)J Recombination

From the discovery of the *RAG* genes on, investigators have suspected that V(D)J recombination may be the result of the landing of a transposable genetic element (a “jumping gene” or transposon) into the vertebrate genome. The clues were many. Firstly, the compact organization of the *RAG* locus resembles a transposable element (Schatz et al. 1989). Secondly, RAGs cut the DNA after binding RSSs throughout the BCR and TCR loci (Gellert 2002). RSSs resemble the ends of other transposable elements. Biochemically, the reaction shares characteristics with enzymes found in other transposable elements (Gellert 2002). Finally, these genes appear abruptly in evolution: they are present in the jawed vertebrates (like the shark), but not in more ancient organisms (Schluter et al. 1999).

A current model is that an ancient transposon containing the *RAG* genes flanked by RSS ends “jumped” into an area of the vertebrate lineage containing a primordial antigen receptor gene, separating it into pieces. When the transposon lifted off, it left the RSSs behind. Multiple rounds of transposition and duplication eventually gave rise to our TCR and BCR loci.

Mechanistically, transposition could happen even now, if the DNA segment containing the SJs is not ligated into a minicircle. These unsealed SJs are able to insert into heterologous sequences in vitro, and if they are allowed to float free in the cell, they have the potential to invade the genome. This phenomenon could give rise to certain types of chromosomal translocations prevalent in some types of B- and T-cell cancers (Shih et al. 2002). Generally, repair processes in the cell actively suppress this phenomenon by rapidly ligating SJs.

## Conclusion

V(D)J recombination is absolutely crucial for the adaptive immune response. In its absence, our immune system is compromised. When it is not properly controlled, it gives rise to chromosomal translocations and B-and T-cell cancers. The elucidation of all steps of the reaction and attempts to understand exactly how these steps are regulated to avoid disastrous side effects are areas of study that have occupied researchers in the past and will continue to do so in the future. 

## Abbreviations

BCR: B-cell receptor
C: constant
CJ: coding joint
D: diversity
DNA-PKcs[RRK1]: DNA protein kinase
H: heavy
J: joining
L: light
RAG: recombination-activating gene
RSS: recombination signal sequence
SCID: severe combined immunodeficiency syndrome
SJ: signal joint
TCR: T-cell receptor
V: variable.
